# PD-L1 aptamer-functionalized degradable hafnium oxide nanoparticles for near infrared-II diagnostic imaging and radiosensitization

**DOI:** 10.3389/fbioe.2023.1224339

**Published:** 2023-06-07

**Authors:** Min Wei, Xiao Shen, Xueqi Fan, Jiwei Li, Jingwen Bai

**Affiliations:** ^1^ Cancer Center and Department of Breast and Thyroid Surgery, Xiang’an Hospital of Xiamen University, School of Medicine, Xiamen University, Xiamen, China; ^2^ Fujian Key Laboratory of Precision Diagnosis and Treatment in Breast Cancer, Xiang’an Hospital of Xiamen University, School of Medicine, Xiamen University, Xiamen, China; ^3^ Xiamen Key Laboratory of Endocrine-Related Cancer Precision Medicine, Xiang’an Hospital of Xiamen University, School of Medicine, Xiamen University, Xiamen, China; ^4^ Xiamen Research Center of Clinical Medicine in Breast and Thyroid Cancers, Xiang’an Hospital of Xiamen University, School of Medicine, Xiamen University, Xiamen, China; ^5^ Cancer Research Center of Xiamen University, School of Medicine, Xiamen University, Xiamen, China; ^6^ Department of Respiratory, Critical Care and Sleep Medicine, Xiang’an Hospital of Xiamen University, School of Medicine, Xiamen University, Xiamen, China; ^7^ Department of Oncology, Xiang’an Hospital of Xiamen University, School of Medicine, Xiamen University, Xiamen, China

**Keywords:** NIR-II fluorescence imaging, PD-L1 aptamer, checkpoint blockade immunotherapy, radiation therapy, tumor treatment

## Abstract

Immune checkpoint blockade is now recognized as a paradigm-shifting cancer therapeutic strategy, whereas there remains difficulty in accurately predicting immunotherapy efficacy by PD-L1 expression. In addition, radiotherapy for cancer patients faces the problem of insufficient dose of radiotherapy at the tumor site while which have been not tolerated by normal tissues. In this study, we created PD-L1 aptamer-anchored spherical nucleic acids (SNAs) with a shell made of PD-L1 aptamer and indocyanine green (ICG) embedded in a mesoporous hafnium oxide nanoparticle core (Hf@ICG-Apt). Upon low pH irradiation in the tumor sites, the nano-system enabled the release of ICG in the high PD-L1 expression tumor to develop a high tumor-to-background ratio of 7.97 ± 0.76 and enhanced the ICG tumor retention to more than 48 h. Moreover, Hf@ICG-Apt improved radiation therapy (RT) when combined with radiation. Notably, Hf@ICG-Apt showed scarcely any systemic toxicity *in vivo*. Overall, this research offered a novel approach for applying reliable monitoring of PD-L1 expression and localization and robust RT sensitization against cancer with good biosafety.

## Introduction

Immunotherapy, especially checkpoint blockade immunotherapy (CBI), has shown a great curative effect in numerous cancers. Immune checkpoint drugs that target PD-1/PD-L1 have improved patient clinical outcomes in a number of advanced tumor forms, enabling a substantial advancement in cancer treatment (Yarchoan et al., 2017). However, due to the poor objective remission rate, there is an ongoing debate over the procedures for selecting patients who are eligible to take anti-PD-1/PD-L1 medications (Qiu, 2020).

Since the PD-L1-positive subgroup patients benefit the most from undergoing anti-PD-1/PD-L1 therapy (Leighl et al., 2019; Kwapisz, 2021), PD-L1 immunohistochemistry (IHC) has been approved by the Food and Drug Administration (FDA) to assess CBI response in patients with a variety of malignancies, including breast cancer, non-small cell lung cancer, and melanoma (Brody et al., 2017; Kwapisz, 2021; Deng et al., 2022; Wolchok et al., 2022). While several studies found that patients with low or no PD-L1 expression level had outcomes similar to those of the group with high PD-L1 expression and the threshold value for PD-L1 was from 1% to 50% (Ancevski Hunter et al., 2018). The difficulty of accurately predicting immunotherapy efficacy by PD-L1 biomarker is a result of a number of factors, including the existence of multiple antibodies for PD-L1 IHC detection with incomparable sensitivity, the different sensitivity of PD-L1 detection platforms, and changes in PD-L1 expression after immunotherapy, surgery, or neoadjuvant chemotherapy (Zhao et al., 2022). It is desirable to develop a novel method to dynamically and intuitively identify changes in PD-L1 expression levels in real time, as this might help to ensure the consistency of PD-L1 measurement findings and contribute to the precise prediction of immunotherapy response.

Due to lower light scattering by tissue and greater penetration depth, optical imaging in the second near-infrared window (NIR-II, 1,000–1800 nm) has a considerable advantage over NIR-I imaging (NIR-I, 700–900 nm) (Hu et al., 2020; Xie et al., 2022). Due to a higher contrast-to-noise ratio of deep structures than in the NIR-I window, indocyanine green (ICG), an FDA-approved substance, has recently been demonstrated to have fluorescent properties in the NIR-II window and is frequently used in several clinical trials for precise surgery and indicating the tumor location in real time (Wu et al., 2022). ICG’s aqueous instability and quick clearance due to the rapid binding to serum proteins limit its application for medical diagnostics (Li and Smith, 2021). Hence, combining ICG with nanocarriers with tumor targeting properties can improve the tumor targeting and retention ability, thus illuminating tumor tissues in real time.

Aptamers are single-stranded DNA or RNA molecules that have undergone Systematic Evolution of Ligands by Exponential Enrichment (SELEX) screening which are referred to as “artificial antibody surrogates” (Ellington and Szostak, 1990). They have distinctive 3D architectures that allow them to precisely bind to cognate molecular targets (Morozov et al., 2021). Due to their ease of modification, minimal batch-to-batch fluctuation, and generally low immunogenicity, aptamers resemble antibodies (Zhang et al., 2023). However, nucleases quickly cleavage aptamers when administered intravenously (Ashraf et al., 2022). Spherical nucleic acids (SNAs), which have a thick shell of highly aligned nucleic acids surrounding a core of nanoparticles (Cutler et al., 2012), have been extensively used to address the aforementioned problem, including mesoporous silica, metal-organic frameworks, liposomes, and so on (Xie et al., 2019; Kim et al., 2022; Zhang et al., 2023). However, core composition alteration is still in its infancy and has poor biocompatibility. Hafnium oxide nanoparticles have been provided in sarcomas, pancreatic ductal adenocarcinoma, and squamous cell carcinoma with a high safety (Bonvalot et al., 2019; Hoffmann et al., 2021; Bagley et al., 2022). Thus, utilizing hafnium oxide combined with aptamers is a potential SNA for clinical translation.

Radiation therapy (RT) is an effective way to shrink tumors and extend survival which remains an important component of cancer treatment (Fitzgerald and Simone, 2020). Radiotherapy works for about 50% of cancer patients (Baskar et al., 2012). Unfortunately, the maximal radiation dose will always be constrained due to toxicity to nearby healthy tissue. Apoptosis is the main process that RT initiated when it comes to causing various forms of cell death to have a therapeutic impact (Sia et al., 2020). The high electron density of clinical functionalized hafnium oxide nanoparticles (NBTXR3) makes them highly likely to interact with incoming ionizing radiation, increasing the energy dose deposit within cells (Zhang et al., 2021). NBTXR3 + RT demonstrated clinically significant improvement for people with locally advanced soft tissue sarcoma compared to RT alone (Bonvalot et al., 2019). However, NBTXR3 is a non-degradable nanoparticle and can only be delivered into tumor tissues by local injection (Marill et al., 2014). The construction of degradable hafnium oxide particles to target tumor tissues throughout the body is a promising drug delivery method with improved biosafety.

Herein, we developed a versatile nanoprobe Hf@ICG-Apt allowing for monitoring of PD-L1 expression and localization and RT sensitization. The ICG was contained within the nanopores of the degradable HfO_2_ core of Hf@ICG-Apt, which also included a PD-L1 aptamer shell. Hf@ICG-Apt improved the ICG aqueous solution stability effectively by containing the almost fluorescence intensity with 30 min irritation. Mesoporous HfO_2_ degraded in tumor sites with the low pH trigger and released ICG more effectively in high PD-L1 tumors with the PD-L1 aptamer targeting. Besides, it protected PD-L1 aptamer from degradation. We also demonstrated that Hf@ICG-Apt could act as a radiosensitizer to produce reactive oxygen species which further triggered apoptosis. In sum, the nanoprobe was expected to generate fresh design concepts for tracking the evolution of PD-L1 expression in CBI response and enhancing radiosensitization in the course of cancer therapy.

## Materials and methods

### Synthesis of Hf@ICG-Apt

First, 60 mL of diluted water was used to dissolve 1.5 g of cetyltrimethylammonium bromide (CTAB, Sigma). This solution was then agitated at 60°C for 0.5 h. Triethanolamine (TEA, 0.18 g, Sigma) was subsequently mixed, and the solution was continuously agitated for 0.5 h. Subsequently, after adding 4 mL of tetraethyl orthosilicate (TEOS, Sigma) into cyclohexane (16 mL), the mixture was dropped into the solution above. After 12 h reaction at 60°C, mesoporous silica (MSN) nanoparticles were produced by centrifuging the solution and washing three times with diluted water. Then, 60 mL of diluted water containing 10 mg MSN and 0.075 g hafnium chloride (Aladdin) was stirred at 90°C for 0.5 h. The mixture was then combined with hexamethylenetetramine (0.15 g, Sigma), and stirred for 24 h at 90°C. To obtain hafnium oxide (HfO_2_) nanoparticles, the solution was centrifuged and rinsed with diluted water.

Secondly, HfO_2_ (50 mg) dissolved in 60 mL of anhydrous ethanol and 0.5 mL of (3-aminopropyl) triethoxysilane (APTES, Sigma) was dropped into the solution. After 12 h stirring at 75°C, the solution was centrifuged and HfO_2_-NH_2_ nanoparticles were obtained. Then, to obtain HfO_2_@ICG-NH_2_, 10 mg HfO_2_-NH_2_ and 2 mg ICG were mixed in diluted water and stirred for 4 h.

The 5′-COOH-modified PD-L1 aptamer MJ5C and random sequence aptamer were purchased from Sangong Biotech with sequences reported previously (Huang et al., 2020). N-hydroxysuccinimide (NHS, 35 mg) and 1-ethyl-3-(3-carbodiimide dimethylaminopropyl) (EDC, 46 mg) were blended in 1 mL of PBS. 4 μL solution were added to 100 μL of 5′-COOH-modified MJ5C aptamer (5 μM), and then added to 450 μL of PBS (pH7.4) and stirred for 4 h in 4°C. Then, HfO_2_@ICG-NH_2_ was added to DNase free water and the activated aptamer was added at a 5% w/w ratio of aptamer/nanoparticles and stirred at 4°C for 24 h. Finally, the mixture was centrifuged (10,000 rpm) to remove free aptamer and HfO_2_@ICG-Apt was obtained.

### Characterization

After the centrifugation, all the supernatants were collected and measured by UV-vis spectrometry at 260 nm to determine the free aptamer amount. The aptamer loading efficiency is calculated as (total aptamer-free aptamer)/total aptamer × 100%. The TEM and SEM analysis were conducted by transmission electron microscopy (H-7650, Hitachi, Japan) and scanning electron microscopy (SEM, S-4800, Hitachi, Japan), respectively. The UV-Vis-NIR spectrophotometer (Cary 5,000, Agilent, United States) was used to conduct UV-vis absorbance. Diameter distribution analysis was obtained by dynamic light scattering (DLS, Omni, Brookhaven, United States).

### ICG and Hf@ICG-Apt photostabilities

ICG and Hf@ICG-Apt solution with the same ICG concentration was added into 96 well plates and irritated by an 808 nm laser. The fluorescence was measured by the NIR-II imaging system (DPM, IVFM, China) with a 1,000 nm long pass filter.

### Hf@ICG-Apt degradation

100 mg Hf@ICG-Apt was dispersed in water solutions with pH 6.5 to simulate the tumor environment. A solution with pH 7.4 was used as the control. The degradation of nanoparticles was analyzed by TEM in 0, 3, 6, 12, and 24 h.

### Cytotoxicity

All the cell lines in this study were purchased from the American Type Culture Collection (ATCC, Rockville, USA). 5 × 10^3^ 4T1 cells were seeded for 6 h in 96 well plates. Different concentrations of Hf@ICG-Apt were added into the cell medium and the nanoprobes were incubated with the cell for 12 h. Cell viability was measured by CCK-8 analysis. As for the cell viability measurement for RT sensitization, 5× 10^3^ 4T1-PD-L1 cells were seeded in the 96 well plates and after cell attachment, they incubated with Hf@ICG-Apt for 24 h before 6 Gy X ray. After X ray, the cells were cultured for an additional 12 h before CCK-8 analysis.

### DNase cleavage protection

To create a 1.5% TAE gel solution, 1.5 g of agarose was dissolved in 100 mL of 1 × Tris-Acetate-EDTA buffer (Beyotime) containing 2 mM EDTA. Then the gel solution was boiled for 4 min with 10 μL nucleic acid gel stain added. 0.5 units/μL of DNase I was added into 200 nM aptamer solution and Hf@ICG-Apt nanoprobe solution [(200 nM aptamer)], and then incubated for 4 h. All of the samples were heated over 95°C for 5 min before being used in the gel electrophoresis. After adding 10 μL of sample per gel’s well, 40 min of 150 V electrophoresis was applied. The gel was then observed and captured using NucleoVision imaging equipment while being exposed to UV light.

### Western blot assay and overexpression cell line construction

Western blot Assay was performed as reported previously (Bai et al., 2017). The primary antibody was anti-PD-L1 antibody (ab233482) and anti-GAPDH antibody (ab9485). The second antibody was goat anti-Rabbit (HRP) (ab97051). All the antibodies were purchased from abcam. The stable overexpression cell line 4T1-PD-L1 and control cell line 4T1-NC were constructed with the HBLV-human-CD274 virus (Hanbio) and followed the protocol of Hanbio.

### Animal models

Female BALB/c mice (20–30 g) were purchased and bred at Xiamen University Laboratory Animal Center. All animal research received approval from the institutional animal care and use committee at Xiamen University, which oversees animal protocols. All studies were conducted strictly in conformity with the applicable regulations.

50 μL of 4T1-PD-L1 cells in PBS were subcutaneously injected into the right hind limbs of BALB/c mice to grow breast cancer tumors. A bilateral tumor model was constructed by injecting subcutaneously with 4T1-PD-L1 and 4T1-NC tumor cells into the right and left hind limbs. At a tumor volume of around 50–100 mm^3^, mice were randomized to various treatment groups.

### Fluorescence imaging and biodistribution analysis using NIR-II

Hf@ICG-Apt and Hf@ICG-Rs were intravenously injected into 4T1-PD-L1 tumor-bearing mice with an equivalent ICG dosage of 1 mg/kg. All the mice were imaged using DPM NIR-II *in vivo* imaging system (IVFM, China) at different time points from 1 h to 96 h after injection. Bilateral tumor mice were sacrificed and their fresh muscles, main organs, and tumors were harvested for NIR-II imaging after injecting Hf@ICG-Apt and Hf@ICG-Rs for 24 h.

### Cell RT sensitization analysis

In the 12-well plate, 1 × 10^5^ 4T1-PD-L1 cells were planted for 6 h. After incubation with Hf@ICG-Apt for 8 h, cells were irradiated with 6 Gy and then were incubated at 37°C for 2 days. For live/dead cell staining analysis, cells were stained for 30 min with Calcein-AM and PI (Beyotime), then detected by confocal laser scanning microscope (CLSM). For live/dead cell staining analysis, cells were stained with Annexin-V FITC/7AAD (Procell) and analyzed by a flow cytometer (Cytoflow, United States). For the cell clone formation test, 4T1-PD-L1 cells were planted in a 6-well plate at a density of 1,000 cells per well for 6 h before being exposed to 6 Gy of radiation. Cells were stained with Crystal Violet Staining Solution (Beyotime) for 10 min after being cultured at 37°C for 14 days, and then they were rinsed with water.

### Evaluation of cellular ROS generation

DCFH-DA (CA1410, Solarbio) was employed for the detection of intracellular ROS. In the 12-well plate, 1 × 10^5^ 4T1-PD-L1 cells were planted for 6 h. The cells were incubated with Hf@ICG-Apt for 8 h and DCFH-DA was added for 20 min before irradiation (6 Gy). The cells were immediately examined using CLSM.

### RT sensitization in 4T1-PD-L1 tumor bearing mice

4T1-PD-L1 tumor-bearing mice with average tumor volume about 50–100 mm^3^ were randomly divided into 4 groups (*n* = 5): PBS, Hf@ICG-Apt, RT (6 Gy), and Hf@ICG-Apt + RT (6 Gy). Each injection contained a dosage of Hf@ICG-Apt equal to 1 mg/kg ([ICG]). The irradiation was performed on the 2nd day and the 8th day. The Hf@ICG-Apt was injected on the 1st day and the 7th day. Throughout the whole experiment, the mouse’s body weight and tumor volume were measured every 3 days. The following formula was used to calculate the tumor volume: 1/2 × width^2^ × length.

### Evaluation of Hf@ICG-Apt biosafety

Hf@ICG-Apt was injected into the healthy BALB/c mouse on day 1 and day 7. The major organs were fixed and sliced for H&E staining. Mice’s blood was collected to measure hematological parameters and blood biochemical parameters.

### Statistical analysis

In order to do the statistical analysis, GraphPad Prism Software was used. One-way analyses of variance (ANOVA) or student’s t-tests were used to prove the statistical significance of the data, which are reported as means ± standard error of the mean (SEM). Kaplan-Meier analysis and a log-rank test with Bonferroni correction were used to measure mouse survival. When *p values <0.05, differences were deemed to be significant.

## Results and discussion

### Characterization of Hf@ICG-Apt

**FIGURE 1:**
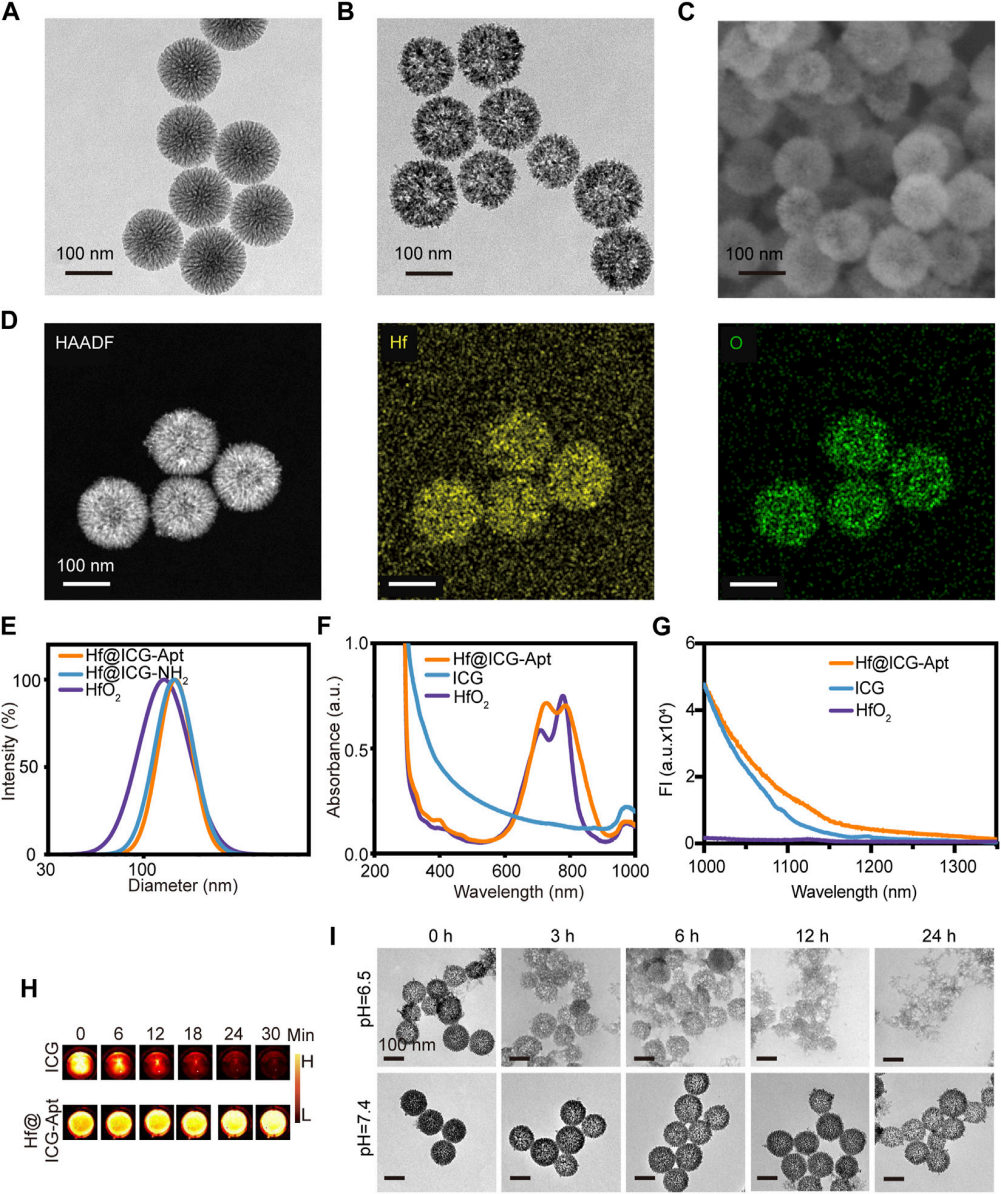
Characterization of nanoprobes. TEM image of MSN **(A)** and Hf@ICG-Apt **(B)**. **(C)** SEM image of Hf@ICG-Apt. **(D)** HAADF and elemental Hf/O mapping images of Hf@ICG-Apt. **(E)** Diameters of HfO_2_, Hf@ICG-NH_2_, and Hf@ICG-Apt. **(F)** UV-vis absorption spectra of HfO_2_, ICG, and Hf@ICG-Apt. **(G)** NIR-II emission spectra of HfO_2_, ICG, and Hf@ICG-Apt. **(H)** ICG and Hf@ICG-Apt NIR-II fluorescence pictures were taken while being continuously irritated by an 808 nm laser. **(I)** Degradation images were observed in different time points by TEM when Hf@ICG-Apt dispersed in water solutions with pH 7.4 or pH 6.5.

In this study, mesoporous silica nanoparticles (MSN) were synthesized by oil/water reaction firstly as the template. TEM image revealed that the MSN nanoparticles were well dispersed with the uniform particle size of ∼130 nm (Figure 1A). Then HfO_2_ was synthesized with hexamethylenetetramine as the reducing agent. After ICG was packaged in HfO_2_ in a water solution with stirring, aptamer PD-L1 functionalized Hf@ICG-Apt was prepared by an EDC/NHS reaction. When studied by TEM and SEM, the resulting Hf@ICG-Apt maintained a spherical shape with a smooth surface, as illustrated in Figures 1B, C. Since the mesopores were smaller and denser the closer to the center, the HfO_2_ synthesized by reverse replication had larger and sparser holes in the center allowing for more ICG loading. The element distribution of Hf@ICG-Apt conducted by a high-angle annular dark-field (HAADF) microscopy elemental mapping analysis confirmed that elements Hf almost consisted with O, proving the successful HfO_2_ construction (Figure 1D).

According to dynamic light scattering (DLS) analysis, after amino modification, nanoparticle diameter increased from 130 nm to 145.31 nm, and it further increased to 151.80 nm after the aptamer PD-L1 was surface attached (Figure 1E). UV–vis-NIR absorption spectrum analysis disclosed that the Hf@ICG-Apt had a similar peak of 781 nm with ICG in 797 nm, demonstrating successful ICG loading in the mesoporous silica (Figure 1F). Zeta potential analysis found that the charge of the Hf@ICG-Apt changed from highly negative (−25.18 ± 2.98 mV) to highly positive (24.76 ± 2.41 mV) after surface functionalization with the amino group, and it decreased to 8.66 ± 2.06 mV ICG loading and aptamer PD-L1 anchoring (Supplementary Figure S1), which implied successful ICG encapsulating and DNA modification. Then, the NIR-II spectral characteristics of Hf@ICG-Apt were verified under the 808 nm laser illumination, which was consistent with ICG, both possessing a tail peak in the NIR-II window (Figure 1G). Each particle had 12.82 nmol/mg of PD-L1 aptamer loading content, as measured by UV-vis spectrometry analysis.

Since ICG could be quenched with continuous laser irritation in an aqueous solution, we verified whether encapsulating ICG into HfO_2_ would be effective in enhancing its stability. The ICG and Hf@ICG-Apt in the 96-well plate were continuously irradiated with an 808 laser (Figure 1H). After 30 min, the ICG solution underwent fluorescence quenching, while the fluorescence intensity in Hf@ICG-Apt remained almost the same (Supplementary Figure S2). This indicated that Hf@ICG-Apt effectively improved the fluorescence stability of ICG, providing a basis for observing PD-L1 expression by fluorescence in subsequent biological applications.

Next, the pH-triggered degradation of our nanoparticle Hf@ICG-Apt was analyzed to simulate the tumor environment. Under normal physical conditions, it appeared to be extremely stable and still kept its spherical structure. Within the low pH environment (pH 6.5), the mesoporous framework became looser at 3 h (Figure 1I). At 6 h, a small part of nanoparticles disintegrated into small fragments and more nanoparticles disintegrated at 12 h. Finally, all the nanoparticles degraded into tiny HfO_2_ fragments at 24 h for ICG cargo release, illustrating the ability of Hf@ICG-Apt to release ICG in tumor tissue.

### Cellular uptake of Hf@ICG-Apt

**FIGURE 2:**
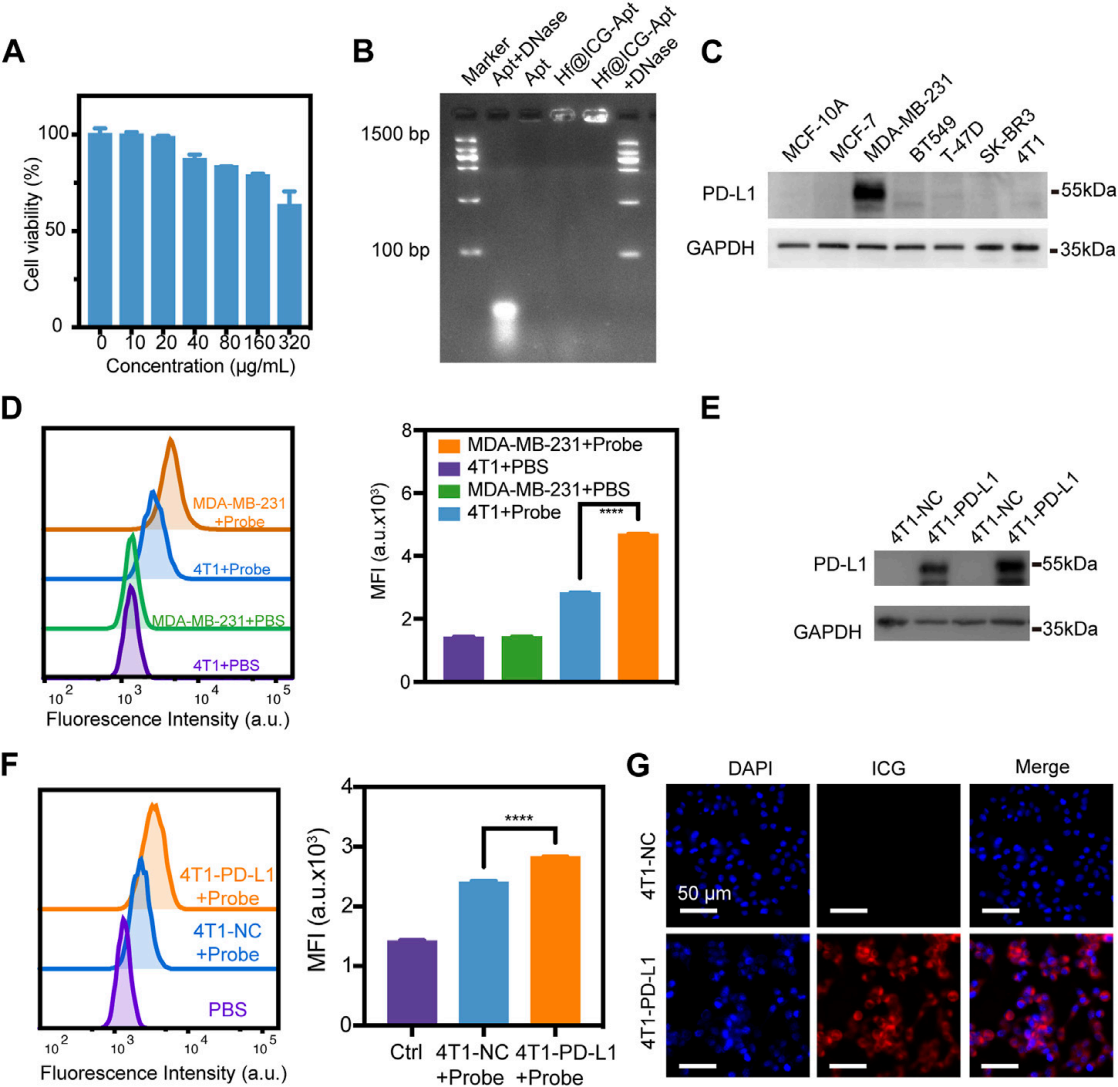
Cellular uptake of nanoprobes. **(A)** Analysis of the cell viability of 4T1 cells after Hf@ICG-Apt incubation at various doses. **(B)** Gel electrophoresis picture for the Hf@ICG-Apt enzymatic cleavage protection test. **(C)** Western blot analysis of PD-L1 expression level in normal breast cell MCF-10A and several breast cancer cell lines. **(D)** Flow cytometry analysis of 4T1 and MDA-MB-231 cell lines incubated with Hf@ICG-Apt (Probe) and the corresponding quantitative statistics of mean fluorescence intensity (MFI). **(E)** Western blot analysis of PD-L1 expression level in stable breast cancer cell 4T1-NC and 4T1-PD-L1. **(F)** Flow cytometry results of 4T1-NC and 4T1-PD-L1 cells incubated with Hf@ICG-Apt (Probe) and the corresponding quantitative statistics of MFI. **(G)** CLSM analysis of 4T1-NC and 4T1-PD-L1 cells incubated with Hf@ICG-Apt (Probe) for 4 h.

The low biotoxicity is the basis for the clinical application of the probe, so we first conducted CCK-8 experiments. After incubation with Hf@ICG-Apt at various concentrations (up to 320 μg/mL), no noticeable reduction in the viability of the cells was found, indicating hardly any cytotoxicity of Hf@ICG-Apt (Figure 2A). Gel electrophoresis was used to investigate the nuclease cleavage protection of our probe. Since degraded DNA fragments would migrate further compared with the original aptamer and be marked by the DNA ladder, we could merely visualize aptamer only and aptamer anchored on Hf@ICG if the protection existed. The free PD-L1 aptamer was digested upon incubation with the nuclease (DNase I) for 4 h showing a vivid migration. While there was no evident enzymatic hydrolysis of the PD-L1 aptamer in the presence of Hf@ICG, only showing a bright band (Figure 2B). The results indicated that single-stranded DNA was possibly quickly and effectively modified on Hf@ICG. Additionally, the DNA was tightly bound to the hafnium oxide surface, preventing DNase from coming in contact with the DNA molecules.

Subsequently, western blot (WB) was utilized to compare the expression of PD-L1 in breast cancer cell lines with the normal breast cell MCF-10A. The results showed that MDA-MB-231 cells had higher levels of PD-L1 expression (Figure 2C). Then, using a flow cytometry test, the PD-L1-specificity maintained by Hf@ICG-Apt was examined. Hf@ICG-Apt (Probe) was applied to the 4T1 cells and MDA-MB-231 cells for 4 h, and the MDA-MB-231 cells’ mean fluorescence intensity (MFI) was greater than that of the 4T1 cells’ (Figure 2D). Further, the 4T1 cell line stable expressing PD-L1 protein (4T1-PD-L1) was established and verified by WB (Figure 2E). After incubating the 4T1-NC and 4T1-PD-L1 cells with Hf@ICG-Apt (Probe), the MFI of the 4T1-PD-L1 cells was proved to be higher than that of the 4T1-NC cells through flow cytometry, indicating the effective cell target ability of Hf@ICG-Apt (Figure 2F). When analyzed by a confocal laser microscope (CLSM), the results showed that the probe was more effectively taken up into the cytoplasm of the 4T1-PD-L1 (Figure 2G). Hence, the above results demonstrated the specific recognition ability with the highly PD-L1-expressing MDA-MB-231 and 4T1-PD-L1 tumor cells.

### PD-L1 targeting NIR-II fluorescence imaging and biodistribution of Hf@ICG-Apt

**FIGURE 3:**
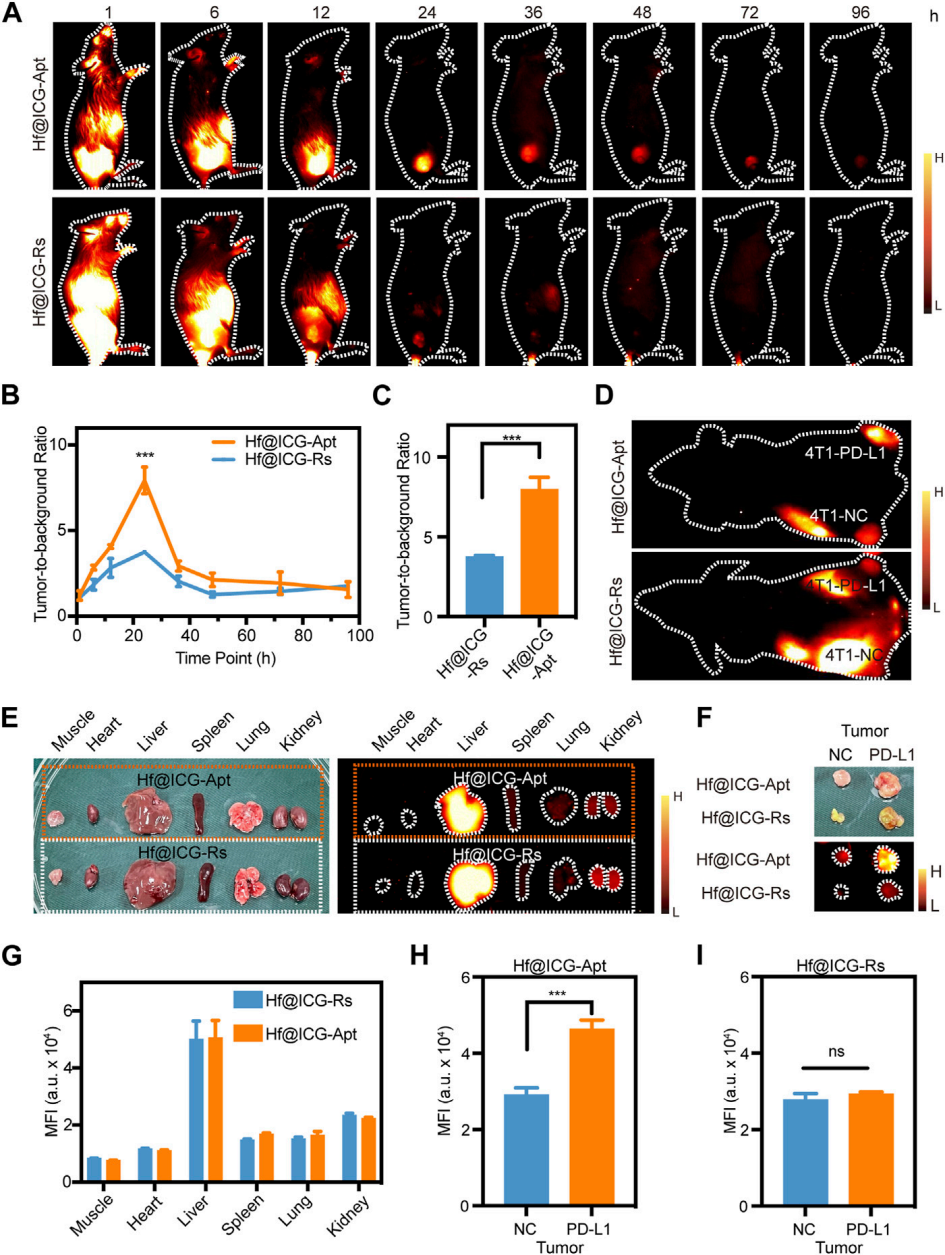
PD-L1 targeting NIR-II fluorescence imaging and biodistribution of Hf@ICG-Apt. **(A)** NIR-II fluorescence images of 4T1-PD-L1 mouse injected with Hf@ICG-Apt and Hf@ICG-Rs. **(B)** TBR analysis of 4T1-PD-L1 mouse injected with Hf@ICG-Apt and Hf@ICG-Rs at various time points. **(C)** TBR analysis of 4T1-PD-L1 mouse injected with Hf@ICG-Apt and Hf@ICG-Rs after 24 h. **(D)** NIR-II fluorescence images of 4T1-NC/PDL1 bilateral tumor mouse injected with Hf@ICG-Apt and Hf@ICG-Rs. **(E)** Pictures and NIR-II fluorescence images in muscles and organs of 4T1-NC/PDL1 bilateral tumor mouse. **(F)** Pictures and NIR-II fluorescence images in tumors of 4T1-NC/PDL1 bilateral tumor mouse. **(G)** The corresponding quantitative statistics of MFI of **(E)**. **(H,I)** The corresponding quantitative statistics of MFI of **(F)**.

To verify the feasibility of Hf@ICG-Apt for highly PD-L1 expressed tumor targeting, 4T1-PD-L1 tumor-bearing mice were injected with Hf@ICG-Apt, and Hf@ICG-Rs (which contains a random sequence rather than the PD-L1 aptamer sequence). NIR-II fluorescence images demonstrated only low signals discernible in the Hf@ICG-Rs group until 72 h (Figure 3A) and the best tumor-to-background ratio (TNR) of 3.74 ± 0.1 at 24 h (Figures 3B, C). However, the Hf@ICG-Apt group gave an obvious ICG signal in the tumor, starting from 24 to 72 h (Figure 3A), with a maximum TNR at 24 h post-injection (7.97 ± 0.76) (Figures 3B, C). The above results demonstrated the advantages garnered by Hf@ICG-Apt in PD-L1-specific targeting and localization.

Next, we used a bilateral tumor model to verify whether Hf@ICG-Apt aggregated differently in tumor tissues with various PD-L1 expression levels *in vivo*. Each BALB/c mouse had a 4T1-NC tumor in the left hind limb and a 4T1-PD-L1 tumor in the right hind limb and was performed with Hf@ICG-Apt or Hf@ICG-Rs intravenously. An obvious MFI difference in bilateral tumors was observed with NIR-II imaging. Of note, the Hf@ICG-Apt-injected mouse displayed a stronger NIR-II fluorescence level than the Hf@ICG-Rs-injected mouse 24 h post-injection (Figure 3D). At the same time, the mouse in two groups was sacrificed and the biodistribution of probes was studied. *Ex vivo* imaging results of excised organs indicated the same organ metabolism pattern (Figures 3E, G). The fluorescence intensities of tumors confirmed enhanced accumulation of Hf@ICG-Apt compared with Hf@ICG-Rs (Figures 3F, H, I). The above results together proved that fabrication with PD-L1 aptamer anchored Hf@ICG could increase the PD-L1 highly expressed tumor recognition ability and thus enabled to distinguish PD-L1 high and low expression tumors non-invasively by NIR-II fluorescence.

### *In vitro* radiation therapy sensitization

Excited by the good targeting performance of the Hf@ICG-Apt probe, considering that the Hf could generate massive reactive oxygen species (ROS) which is a key factor in radiation therapy (RT), the investigation of its therapeutic efficacy of radiation sensitization was performed in 4T1-PD-L1 tumor cells.

**FIGURE 4:**
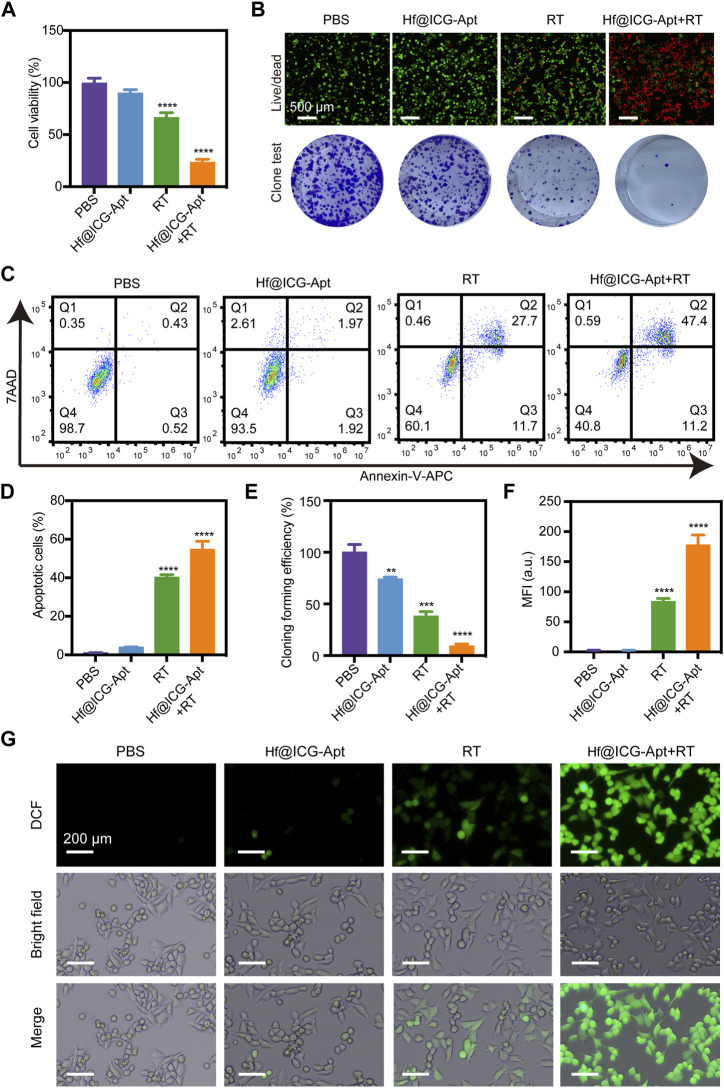
*In Vitro* radiation therapy sensitization. **(A)** Cell viability of 4T1-PD-L1 cells after different treatments. **(B)** Live/dead analysis and clone forming test of 4T1-PD-L1 cells after different treatments. **(C)** Apoptosis analysis of 4T1-PD-L1 cells after different treatments. **(D)** The corresponding quantitative statistics of apoptotic cells in **(C)**. **(E)** The corresponding quantitative statistics of cloning forming efficiency in **(B)**. **(F)** The corresponding quantitative statistics of MFI in **(G)**. **(G)** CLSM images of ROS generation in 4T1-PD-L1 cells with various treatments.

In the CCK-8 assay, when combined with RT, Hf@ICG-Apt brought the most significant decrease in cell viability (Figure 4A), indicating a potent RT sensitization effect. Then, live/dead cell staining and clone formation analysis were conducted to identify the *in vitro* cell-killing ability of Hf@ICG-Apt. As seen in Figure 4B, negligible dead cells were revealed when treated with Hf@ICG-Apt, whereas most cell death occurred in the Hf@ICG-Apt + RT group showing an obvious red fluorescence (Figure 4B). Similarly, the clone formation test also figured out the most effective treatment was Hf@ICG-Apt + RT owing to the least cell cloning formation after 14 days (Figures 4B, E). Next, the apoptosis cells were measured with annexin-V/7AAD staining assay, and the results showed that Hf@ICG-Apt + RT induced 54.7% apoptosis cells which were remarkably higher than the RT group (40.3%) (Figures 4C, D), inducing effective cell killing. Subsequently, we conducted the reactive oxygen species (ROS) detective assay with 2′,7′-dichlorofluorescein diacetate (DCFH-DA). Without being exposed to radiation, Hf@ICG-Apt displayed weak green fluorescence. However, following exposure made the fluorescence signal became robust (Figures 4F, G), indicating that Hf@ICG-Apt retained the responsive ROS production capacity with the Hf element.

### *In vivo* radiation therapy sensitization

**FIGURE 5:**
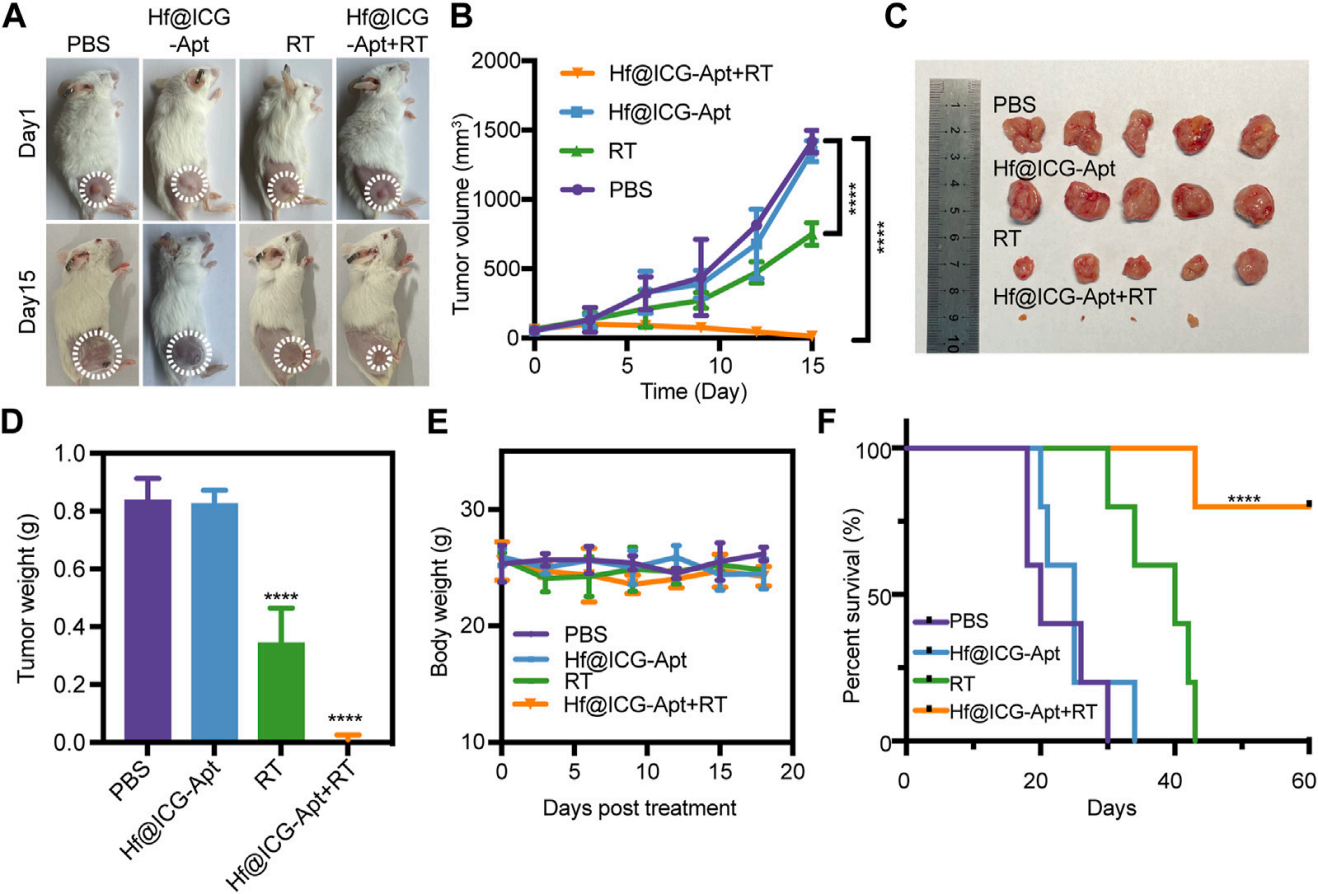
*In vivo* evaluation of radiation therapy efficacy. **(A)** Pictures of 4T1-PD-L1 tumor bearing mice on day 1 and day 15 in different treatment groups. **(B)** Inhibition of tumor growth in tumor-bearing mice treated with different formulations, followed by observation of the tumor size over a period of 15 days (*n* = 5). **(C)** Picture of tumor from breast cancer-bearing mice receiving various therapeutic treatments (*n* = 5). **(D)** The tumor weight of tumor-bearing mice. **(E)** The tumor-bearing mouse’s body weight after undergoing various therapeutic procedures. **(F)** Kaplan-Meier survival curves of the tumor-bearing mice undergoing different interventions after 60 days.

The therapeutic impact was subsequently investigated *in vivo* on the basis of the promising RT sensitization effectiveness of Hf@ICG-Apt in breast cancer cells above. 4T1-PD-L1 tumor bearing mouse were randomly divided into 4 groups with different treatments: 1) PBS; 2) Hf@ICG-Apt; 3) RT (6 Gy); 4) Hf@ICG-Apt + RT (6 Gy). Mice received a total of two treatments, administered once every 7 days. To assess the therapeutic effectiveness of various therapies, tumor volume was dynamically assessed (Figure 5A). Analysis showed that tumors expanded quickly following treatment with PBS but were partially suppressed by RT (Figure 5B). Interestingly, for the group of mice injected Hf@ICG-Apt combined RT, tumors were greatly suppressed after the treatment, compared with those without irradiation or RT alone (Figures 5B, C). Moreover, the average tumor weight of the combined treatment group was also the least (Figure 5D), identifying that Hf@ICG-Apt + RT treatment potentially lessens the burden of currently existing tumors. Meanwhile, little body weight reduction was seen while receiving therapy (Figure 5E). Among all the 4 groups with different treatments, Hf@ICG-Apt + RT demonstrated the most effective treatment and had the highest survival rate (Figure 5F).

### Hf@ICG-Apt biosafety

**FIGURE 6:**
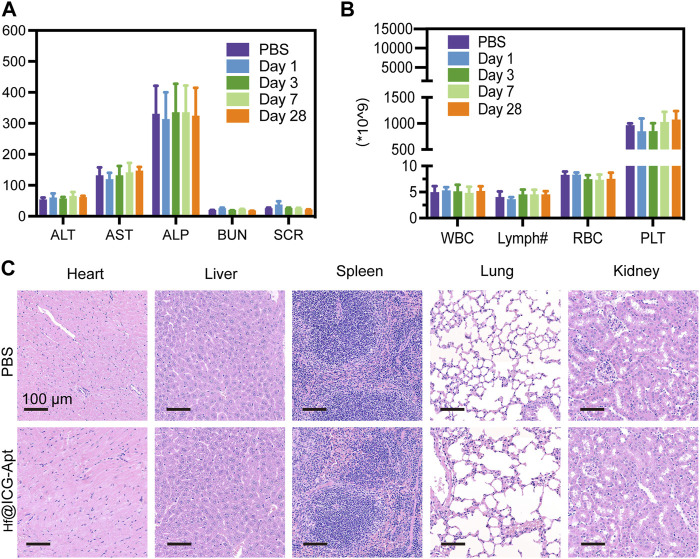
Biosafety of Hf@ICG-Apt. **(A)** Hepatic and renal function change during the treatment. **(B)** Blood biochemical parameters changes during the treatment. **(C)** H&E staining images of the main organs on day 28.

We eventually carried out the systematic toxicity investigations of our probe, which were inspired by the PD-L1 tumor targeting described above under NIR-II fluorescence imaging and RT sensitization effect. First, after 28 days post-injection, the major hematological parameters and blood biochemical parameters were monitored. The results suggested that these parameters of our nanoprobe showed low changes when compared to the PBS group (Figures 6A, B). The histological examinations of the major organs that were randomly removed from the chosen BALB/c mice have subsequently proven that the Hf@ICG-Apt had such a nontoxic quality since no consistent adverse effects to the major organs could be seen (Figure 6C). The aforementioned findings supported the possibility of future clinical translations by indicating that the dose regimens were well-tolerated and that this nano-system had high biocompatibility. Since there were some fluorescence signals in lungs in Figure 3E, the prolonged toxicity, in-depth impacts on metabolism, particularly in the lungs still need to be examined.

## Conclusion

In conclusion, we have constructed a degradable mesoporous hafnium-based aptamer PD-L1 modified SNA nano-system (Hf@ICG-Apt) for diagnosing high PD-L1 expression tumor with NIR-II imaging and radiotherapy sensitization. The nanoprobe enhanced the stability of ICG in aqueous solution, protected aptamer PD-L1 from nuclease degradation, and improved their accumulation in the high PD-L1 expressed tumor sites. Of note, Hf@ICG-Apt could figure out the PD-L1 expression differences with NIR-II imaging both *in vivo* and *in vitro*. Moreover, Hf@ICG-Apt acted as a radiosensitizer for generating cellular ROS and thus activated RT sensitization via apoptosis. Our nanoplatform may shed light on the development of clinical early diagnostic imaging of PD-L1 alterations in CBI treatment as well as next-generation radiation therapeutic systems.

## Data Availability

The raw data supporting the conclusion of this article will be made available by the authors, without undue reservation.
