# Supplementary material for: PD-L1 aptamer-functionalized degradable hafnium oxide nanoparticles for near infrared-II diagnostic imaging and radiosensitization
Source: Front Bioeng Biotechnol. 2023 Jun 7;11:1224339. doi: 10.3389/fbioe.2023.1224339 (PMC10282151; doi:10.3389/fbioe.2023.1224339)
Supplement: Supplementary file 1 [file DataSheet1.DOCX]

Supplementary Material

PD-L1 Aptamer-Functionalized Degradable Hafnium Oxide Nanoparticles for Near Infrared-II Diagnostic Imaging and Radiosensitization

**Min Wei^1,2,3,4,5^, Xiao Shen^1,2,3,4,5^, Xueqi Fan^1,2,3,4,5^, Jiwei Li^7^, Jingwen Bai^1,2,3,4,5,6*^**

*** Correspondence:** Jingwen Bai: baijingwen666@126.com


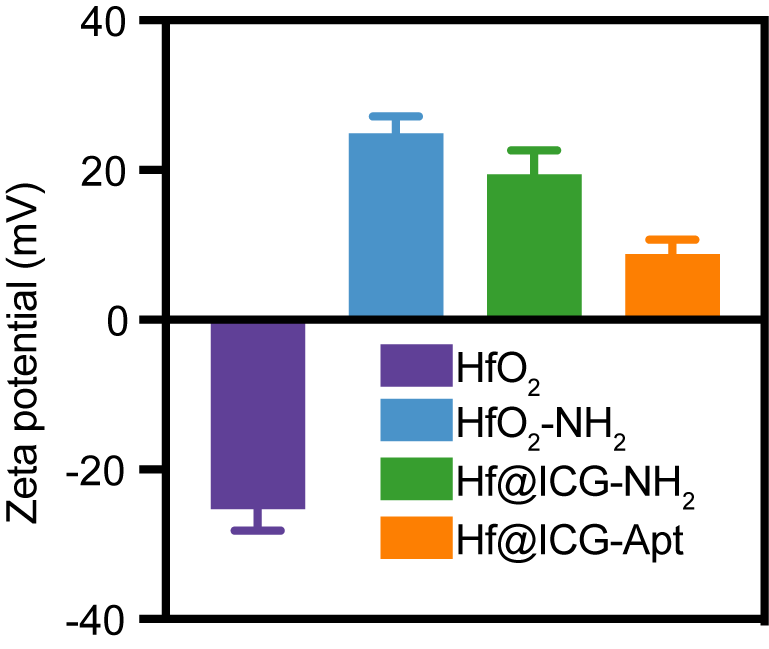


**Supplementary Figure 1.** The zeta potential changes during the synthesis of Hf@ICG-Apt.


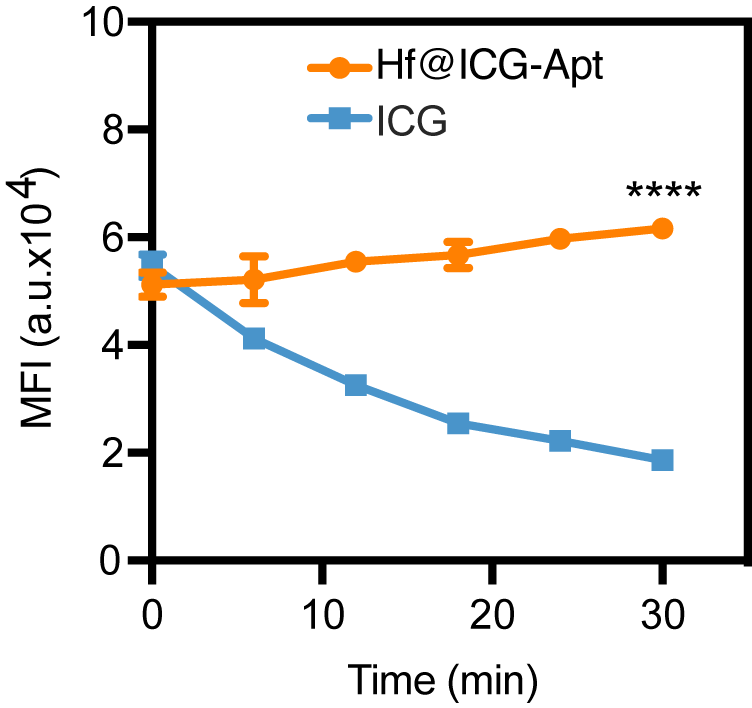


**Supplementary Figure 2.** The corresponding statistics of MFI in figure 1H.
